# The clinical overlap between functional dyspepsia and irritable bowel syndrome based on Rome III criteria

**DOI:** 10.1186/1471-230X-8-43

**Published:** 2008-09-23

**Authors:** AnJiang WANG, XianHua LIAO, LiShou XIONG, Sui PENG, YingLian XIAO, SiChun LIU, PinJin HU, MinHu CHEN

**Affiliations:** 1 Department of Gastroenterology, the First Affiliated Hospital, Sun Yat-Sen University, Guangzhou, PR China

## Abstract

**Background:**

Epidemiological studies suggest considerable overlap between functional dyspepsia (FD) and irritable bowel syndrome (IBS). To date, no surveys have been performed to investigate the clinical overlap between these two disorders using Rome III criteria. Our aim was to investigate the prevalence and risk factors for the overlap of FD and IBS based on Rome III criteria in a large clinical sample.

**Methods:**

Consecutive patients at the general gastroenterology outpatient clinic were requested to complete a self-report questionnaire. FD and IBS were defined by Rome III criteria.

**Results:**

Questionnaires were returned by 3014 patients (52.8% female, 89% response rate). FD-IBS overlap was observed in 5.0% of the patients, while 15.2% and 10.9% of the patients were classified as FD alone and IBS alone, respectively. Compared with non-IBS patients, the odds ratio of having FD among IBS patients was 2.09 (95% CI: 1.68–2.59). Patients with FD-IBS overlap had higher severity scores for the postprandial fullness symptom (2.35 ± 1.49 vs. 1.72 ± 1.59, P < 0.001) and overall FD symptom (6.65 ± 2.88 vs. 5.82 ± 2.76, P = 0.002) than those with FD alone. The only independent risk factor for FD-IBS overlap vs. FD alone was the presence of postprandial fullness symptom (OR 2.67, 95% CI: 1.34–5.31).

**Conclusion:**

Clinical overlap of FD and IBS according to Rome III criteria is very common. One risk factor for FD-IBS overlap is the presence of postprandial fullness symptom. This study provides clues for future pathophysiological studies of FD and IBS.

## Background

A high prevalence of overlap between functional dyspepsia (FD) and irritable bowel syndrome (IBS) has been consistently and universally reported. Epidemiological data suggest that about 13%–87% of patients with either diagnosis fulfill the criteria for the other diagnosis [1]. It is possible that there is a distinct subgroup with overlap of FD and IBS having a generalized rather than a regional disorder of gut with common pathophysiological mechanisms.

Different definitions of FD and IBS have been shown to identify patients with different characteristics. The new definition of FD according to Rome III criteria, which is based on pathophysiological studies and factor analysis, has resulted in a more homogenous selection of patients and more pathophysiologically relevant symptoms. Current activity of these two disorders indicated by Rome III criteria, has changed the symptomatic characteristics of FD and IBS. In addition, the discovery that the presence of IBS does not exclude the diagnosis of any functional gastroduodenal disorder makes the evaluation of overlap between FD and IBS with Rome III criteria more accurate. Therefore, the overlap between FD and IBS according to Rome III criteria may be different from such overlap diagnosed with the former criteria. No studies have been performed to investigate the overlap between FD and IBS according to Rome III criteria. Since most pathophysiological studies enroll subjects from clinics, a study of the clinical overlap of FD and IBS may provide clues and new insights for future pathophysiological studies of both disorders. The aim of the present study was to determine the prevalence and risk factors for the clinical overlap between FD and IBS defined by Rome III criteria in Chinese patients.

## Materials and methods

### Study setting

The setting for this study was the general gastroenterology outpatient clinic of the first affiliated hospital of Sun Yat-sen University in Guangzhou, China. The outpatient clinic is an open access system since it provides primary, secondary and tertiary level care. Patients with any gastrointestinal disorder may seek healthcare at this clinic. Written informed consent was obtained from all the participants. This study was approved by the Ethics Committee of the first affiliated hospital of Sun Yat-sen University.

### Sample size calculation

Based on the 1996 epidemiologic study, the prevalence of FD-IBS overlap in the Guangdong general population was 4% [2]. The result of the pilot study conducted in 1000 consecutive patients at the general gastroenterology outpatient clinic of the first affiliated hospital of Sun Yat-sen University showed that the prevalence of overlap was 6%. Based on sample size calculations according to established formulas [3], the minimum sample size necessary for the current study was found to be 385 participants. As 3014 patients were enrolled in this study, the sample size was sufficient for estimating the prevalence of FD-IBS overlap.

### Subjects and survey methods

Consecutive Chinese patients (age ≥ 18 years) who presented at the general gastroenterology outpatient clinic were requested to fill out a self-report questionnaire. The questionnaires were dispensed to patients by two doctors who did not intervene in the patients' medical management. All patients were requested to complete the questions by marking the appropriate response boxes with ticks. They were requested to consult the two doctors if they did not understand a question in the questionnaire. Those who chose not to complete the questionnaire were requested to return it. Each eligible patient was asked to fill in the questionnaire only once during the survey period. Patients who had major psychotic episodes, mental retardation, dementia, severe visual or hearing abnormalities or other illnesses that might render them unable to complete the questionnaire (e.g., stroke) and those that had used specific drugs, including NSAIDs, steroids or drugs affecting gastric acid secretion or gastrointestinal motility in the 3 months prior to the investigation were excluded.

### Questionnaire

The self-report questionnaire included ① demographics, such as age and gender; ② the Chinese version of the previously validated Rome III diagnostic questionnaire for adult functional dyspepsia and irritable bowel syndrome designed by the Rome committee by which patients were defined as FD or IBS [4]. In the development of the Chinese version of the questionnaire, the original instrument was translated, backtranslated and tested for reproducibility in a sample of 68 patients over a 2 week period. The intra-class correlation coefficient of the translated questionnaire for adult FD and IBS was 0.88 (95% CI: 0.84–0.92). FD patients were classified according to their symptoms as having postprandial distress syndrome (PDS) alone, epigastric pain syndrome (EPS) alone or coexistence of PDS and EPS (PDS+EPS) subtypes. IBS patients were classified, as determined by their predominant stool pattern, to be four subtypes: IBS with constipation (IBS-C), IBS with diarrhea (IBS-D), mixed IBS (IBS-M) and unsubtyped IBS (IBS-U); ③ the severity of four different dyspeptic symptoms (postprandial fullness, early satiation, epigastric pain and epigastric burning) and abdominal pain/discomfort relieved by defecation or associated with an altered bowel habit based on a 6-point scale [5,6] (0, none; 1, very mild, could be easily ignored without effort; 2, mild, could be ignored with effort, but would not influence daily activities; 3, moderate, could not be ignored and occasionally limits daily activities; 4, severe, could not be ignored and often limits concentration on daily activities; 5, very severe, could not be ignored and markedly limits daily activities and often requires rest. An overall FD symptom score was calculated by summing the severity score of each symptom; ④ the frequency of nine different abnormal bowel habits (fewer than three bowel movements per week; more than three bowel movements per day; hard or lumpy stools; loose (mushy) or watery stools; straining during a bowel movement; urgency; feeling of incomplete bowel movement; passing mucus during a bowel movement; abdominal fullness, bloating or swelling) based on a 6-point scale (0, none; 1, < 25% of the time; 2, 25%–50% of the time; 3, 50%–75% of the time; 4, > 75% of the time, but not always; 5, always). An overall IBS abnormal bowel habit score was calculated by summing the frequency score of each symptom. Patients with a history of abdominal or gynecological surgery, gastrointestinal cancer, a documented peptic ulcer disease, ulcerative colitis, Crohn's disease, diabetes mellitus or hyperthyroidism were excluded from diagnosis of FD or IBS.

### Statistical analysis

A Student's *t *test was used to compare the distributions of age and individual symptom score. Distributions of sex, individual symptom and subtype were compared by Pearson's chi-square test. A bivariate logistic regression analysis was carried out to measure the association between FD and IBS. To identity the risk factors for overlap, the statistical analysis involved three steps: description of variation with all potential risk factors for overlap, univariate analysis of the probability of overlap with single independent variables and multivariate analysis using logistic regression. A *P *value of 0.2 in the univariate analysis was chosen as a cutoff point to decide whether a variable could be included in the multivariate logistic regression analysis of the risk of FD-IBS overlap. Odds ratios with 95% confidence intervals (CIs) were computed. All the statistical comparisons were two-sided using the 0.05 significance level. The data were processed, and statistical analysis was performed with a SPSS13.0 program (SPSS Inc., Chicago, IL, USA).

## Results

### Sample characteristics

Of the 3379 eligible patients, 3014 patients completed questionnaires (response rate 89.2%). Overall, 47.2% (1422 patients) of the respondents were male with the mean (± s.d.) age of 43.2 (± 15.9) years and a range of 18–97 years. Among non-respondents, 47.6% were males with a mean (± s.d.) age of 44.0 (± 16.0) with a range of 20–95 years. There was no significant difference between the responders and non-responders with respect to sex or age.

### Demographic and symptomatic characteristics of FD-IBS overlap versus FD alone and versus IBS alone

**Figure 1:**
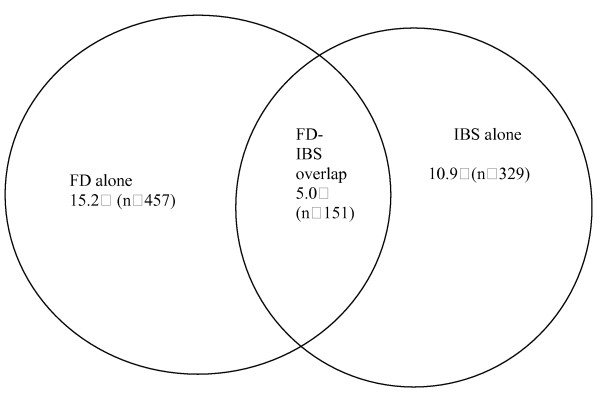
The prevalence of clinical overlap of functional dyspepsia (FD) and irritable bowel syndrome (IBS) (Total number of patients = 3014).

The demographic and symptomatic characteristics of patients with FD-IBS overlap, FD alone and IBS alone are shown in Tables 1 and 2. Overlap between FD and IBS according to Rome III criteria was very common in clinical practice, as summarized in Figure 1; in addition, 24.8% (151/608) of FD patients and 31.5% (151/480) of IBS patients fulfilled the diagnostic criteria of the other disorder. There was no significant difference with respect to sex or age among FD alone, IBS alone and FD-IBS overlap groups. The odds ratio [the ratio of the odds of having IBS among those with FD to the odds of having IBS among those without FD, or the ratio of the odds of having FD among those with IBS to the odds of having FD among those without IBS] was 2.09 (95% CI: 1.68–2.59).

**Table 1:** Comparison of the demographics, FD symptoms and subtypes in the FD-IBS clinical overlap group with those of the FD alone group

	**FD-IBS overlap**	**FD alone**	***P***
	**(n = 151)**	**(n = 457)**	
**Demographics**			
Age (years; $\bar{x}$ ± *s.d*.)	42.91 ± 14.61	42.37 ± 14.84	0.70
Female Gender (%)	79(52.3)	264(57.8)	0.24
**Symptoms (%)**			
(**score**, $\bar{x}$ ± *s.d*.)			
Early satiation	58(38.4)	152(33.3)	0.25
	(1.13 ± 1.52)	(0.95 ± 1.45)	0.21
Postprandial fullness	115(76.2)	266(58.2)	<0.001**
	(2.35 ± 1.49)	(1.72 ± 1.59)	<0.001**
Epigastric pain	113(74.8)	342(74.8)	1.00
	(2.24 ± 1.50)	(2.42 ± 1.58)	0.22
Epigastric burning	49(32.5)	118(25.8)	0.11
	(0.93 ± 1.43)	(0.73 ± 1.33)	0.14
**Overall symptom score**	6.65 ± 2.88	5.82 ± 2.76	0.002*
**Subtypes(%)**			
PDS alone	59(39.1)	164(35.9)	0.48
EPS alone	41(27.2)	183(40.0)	0.004*
PDS+EPS	51(33.8)	110(24.1)	0.02*

FD, functional dyspepsia; IBS, irritable bowel syndrome; PDS, postprandial distress syndrome; EPS, epigastric pain syndrome; PDS+EPS, coexistence of PDS and EPS;

*P < 0.05,**P < 0.001

**Table 2:** Comparison of the demographics, IBS symptoms and subtypes of the FD-IBS clinical overlap group with those of the IBS alone group

	**FD-IBS overlap**	**IBS alone**	***P***
	**(n = 151)**	**(n = 329)**	
**Demographics**			
Age (years; $\bar{x}$ ± *s.d*.)	42.91 ± 14.61	43.63 ± 16.21	0.63
Female Gender (%)	79(52.3)	194(59.0)	0.17
**Severity score of abdominal pain or discomfort**	2.95 ± 0.76	2.87 ± 0.81	0.34
**Symptoms (%)**			
(**score**, $\bar{x}$ ± *s.d*.)			
<3 bowel movements/week	39(25.8)	79(24.0)	0.67
	(1.52 ± 1.05)	(1.46 ± 0.96)	0.53
>3 bowel movements/day	31(20.5)	71(21.6)	0.79
	(1.33 ± 0.79)	(1.48 ± 1.06)	0.08
Hard or lumpy stools	57(37.7)	109(33.1)	0.32
	(1.92 ± 1.37)	(1.75 ± 1.23)	0.19
Loose or watery stools	55(36.4)	134(40.7)	0.37
	(1.73 ± 1.21)	(1.92 ± 1.34)	0.12
Defecation straining	63(41.7)	121(36.8)	0.30
	(2.04 ± 1.47)	(1.91 ± 1.39)	0.36
Urgency	56(37.1)	136(41.3)	0.38
	(1.87 ± 1.34)	(1.98 ± 1.39)	0.39
A feeling of incomplete bowel movement	80(53.0)	156(47.4)	0.26
	(2.26 ± 1.46)	(2.06 ± 1.37)	0.14
Passing mucus	40(26.5)	84(25.5)	0.82
	(1.40 ± 0.83)	(1.46 ± 0.96)	0.52
Abdominal fullness, bloating or swelling	103(68.2)	212(64.4)	0.42
	(2.66 ± 1.49)	(2.54 ± 1.48)	0.42
**Overall score of abnormal bowel habit**	16.74 ± 6.05	16.58 ± 5.05	0.78
**Subtypes (%)**			
IBS-C	43(28.5)	91(27.7)	0.85
IBS-D	41(27.2)	116(35.3)	0.08
IBS-M	14(9.3)	18(5.5)	0.12
IBS-U	53(35.1)	104(31.6)	0.45

FD, functional dyspepsia; IBS, irritable bowel syndrome; IBS-C, IBS with constipation; IBS-D, IBS with diarrhea; IBS-M, mixed IBS; IBS-U, unsubtyped IBS

More patients with PDS alone had IBS than those with EPS alone (26.5% vs. 18.3%, *P *= 0.039). Patients with FD-IBS overlap were more likely to be classified as the PDS+EPS subtype (33.8% vs. 24.1%, *P *= 0.02), had more frequent presence of the postprandial fullness symptom (76.2% vs. 58.2%, *P *< 0.001) and higher severity scores for the postprandial fullness symptom (2.35 ± 1.49 vs. 1.72 ± 1.59, *P *< 0.001) and overall FD symptom (6.65 ± 2.88 vs. 5.82 ± 2.76, *P *= 0.002) than those with FD alone. In addition, they were less likely to be classified as the EPS alone subtype (27.2% vs. 40.0%, *P *= 0.004) compared to patients classified as FD alone (Table 1). Demographic features and patterns of symptom and subtype were not significantly different between patients with FD-IBS overlap and IBS alone (Table 2).

### Potential risk factors for FD-IBS overlap

Table 1 summarizes the comparison of variables in patients with FD-IBS overlap relative to those with FD alone. Of the dichotomously classified potential correlates of the probability of overlap, the presence of postprandial fullness symptom (*P* < 0.001), epigastric burning symptom (*P* = 0.11) and EPS alone subtype (*P* = 0.004) were included in the multivariate logistic regression analysis. Of these, only the presence of postprandial fullness (OR 2.67, 95% CI: 1.34–5.31, *P* = 0.005) had a statistically significant and independent effect on the probability of having overlap in the final logistic model (Table 3).

**Table 3:** Multivariable analysis of risk factors for FD-IBS overlap and FD alone

Potential risk factors	*β*	OR	*P*	95% CI
Postprandial fullness	0.98	2.67	0.005*	1.34–5.31
Epigastric burning	0.38	1.47	0.07	0.97–2.21
EPS alone subtype	0.15	1.16	0.66	0.59–2.29

FD, functional dyspepsia; IBS, irritable bowel syndrome; EPS, epigastric pain syndrome; *P < 0.05

Table 2 summarizes the comparison of variables in patients with FD-IBS overlap relative to those of patients with IBS alone. Male gender (*P* = 0.17) and subtypes of IBS-M (*P* = 0.12) and non-IBS-D (*P* = 0.08) were included in multivariate logistic regression analysis after the univariate analysis. As shown in Table 4, none of these variables were identified as risk factors for FD-IBS clinical overlap vs. IBS alone in the final logistic model.

**Table 4:** Multivariable analysis of risk factors for FD-IBS overlap and IBS alone

Potential risk factors	*β*	OR	*P*	95% CI
Male gender	0.68	1.37	0.11	0.93–2.03
IBS-D subtype	0.64	1.44	0.10	0.93–2.22
IBS-M subtype	0.48	1.62	0.21	0.77–3.40

FD, functional dyspepsia; IBS, irritable bowel syndrome; IBS-D, IBS with diarrhea; IBS-M, mixed IBS;

## Discussion

The results of the present study demonstrate that clinical overlap between FD and IBS according to Rome III criteria is very common and that the association is highly unlikely to be explained by chance. In addition, the presence of postprandial fullness symptom was shown to be a risk factor for FD-IBS overlap.

Epidemiological studies concerning the rate of FD and IBS concurrence have demonstrated a wide range (13–87%) of overlap between these two disorders [1,7-16]. This wide range may be due to differences in these studies such as the varied study populations from different countries and different diagnostic criteria used. In Asia, Shah et al. [15] found that 58% of subjects with IBS had dyspeptic symptoms, while 14% of subjects with dyspeptic symptoms had IBS in a population-based study in Mumbai, India. In the Guangdong province of China, Chen et al. [16] reported that 23.7% of the subjects with dyspeptic symptoms had IBS, which was greater than the proportion of subjects that lacked dyspeptic symptoms and were diagnosed with IBS (6.5%). Importantly, most of these studies limited their investigation to a one-way association between FD and IBS (e.g., FD vs. FD-IBS or IBS vs. FD-IBS) and no study has investigated the clinical overlap between FD and IBS using Rome III criteria. In our study, we observed that the clinical overlap of FD and IBS was very common in patients. The odds ratio was significantly greater than 1.0, which indicated that there was a strong positive association between the two disorders defined by Rome III criteria. Thus, this study provides additional information on the clinical overlap between FD and IBS defined by Rome III criteria in a clinically relevant sample in China.

Some studies [7,17] have found that patients with FD-IBS overlap had more symptoms and subtypes of the dysmotility type. Stanghellini et al. [8] found that patients with predominantly non-painful symptoms, such as postprandial fullness, nausea and vomiting, were more likely to be diagnosed with IBS than patients that predominantly experienced epigastric pain. Our results suggest that more patients with PDS alone overlapped IBS than those with EPS alone and patients with FD-IBS overlap had more presence of postprandial fullness symptom than those with FD alone. These findings support those reported in the above-mentioned studies. In addition, the current study indicates that patients with FD-IBS overlap are more likely to be classified as the PDS+EPS subtype and less likely to be classified as the EPS alone subtype. This finding has not been previously reported. The results presented here should be confirmed and the implications should be further explored in future epidemiological and pathophysiological studies. In particular, our results have shown that the symptom of postprandial fullness was significantly associated with the clinical overlap of FD and IBS. As several studies suggest that the pathophysiological mechanism of FD is associated with a specific symptom or subtype of FD [18-21], we speculate that the mechanism that underlies the symptom of postprandial fullness (such as delayed gastric emptying or impaired accommodation) may play a role in the pathophysiological mechanisms of both FD and IBS.

Many studies have demonstrated that IBS-C patients are more likely to exhibit overlap with FD and tend to have more symptoms of dysmotility type than IBS-D patients [9,10,22]. Pathophysiological research has also shown that about 30% of IBS patients have delayed gastric emptying, which may be observed more frequently in patients with constipation-predominant IBS than in those with diarrhea-predominant IBS [23-28]. Recently, Stanghellini et al. [29] reported that gastric emptying was delayed in IBS patients with concomitant FD, but not in those with IBS alone. However, our study showed that the demographics, subtypes and symptoms of IBS did not differ between patients with IBS-C and IBS-D and that these were not independent risk factors of FD-IBS overlap vs. IBS alone. Compared to previously reported findings, the differences in the findings reported here may be attributed to the different way of subtyping IBS according to Rome III criteria that was performed in our study but not in previous studies. The symptom pattern and pathophysiological mechanisms of similar subtypes defined by different criteria (e.g., constipation-predominant IBS vs. IBS with constipation; diarrhea-predominant IBS vs. IBS with diarrhea) may be distinct.

The severity of symptoms is also associated with pathophysiological mechanisms [1,18-20]. Tack et al. [1] found that patients with FD-IBS overlap, who had a greater prevalence of hypersensitivity to gastric distention, suffered from more severe FD symptoms in general as compared with patients with FD alone. In the present study, we asked patients to rate their symptom severity and frequency on a 6-point scale. Our results indicate that patients with overlap suffered from more severe postprandial fullness symptom and overall FD symptom.

Our study has some limitations. First, all of the patients were from one hospital. However, as the hospital provides primary, secondary and tertiary medical care and since our clinical sample was very large, patients from different levels of care were well represented here. Thus, our study could reflect the clinical overlap between FD and IBS that is likely to be observed at a general gastroenterology outpatient clinic. Our results should be confirmed by future multi-center investigations in larger clinical samples. Second, the diagnosis of patients in our study was not confirmed by objective examinations. But the standardized diagnostic Rome III questionnaire, which has a high sensitivity and specificity for diagnosing functional gastrointestinal disorders, is an efficient diagnostic tool, which facilitates analysis of a large clinically relevant sample and allows for studying the epidemiology of overlap.

To the best of our knowledge, this is the first epidemiological investigation on the prevalence and risk factors of clinical overlap between FD and IBS using Rome III criteria. The standardized questionnaire could permit comparison of our results to those of other studies. Since the doctors in charge of this survey did not intervene with the patients' medical management, the objectivity of questionnaires completed by patients themselves was ensured.

## Conclusion

Clinical overlap of FD and IBS according to Rome III criteria is very common. There is a positive association between these two disorders. Only the presence of postprandial fullness symptom independently predicts FD-IBS overlap versus FD alone. Our results suggest that there is likely to be a common disease process in a subgroup of patients with FD-IBS overlap. These findings may provide some clues and new insights for future pathophysiological studies of FD and IBS defined according to Rome III criteria.

## Competing interests

The authors declare that they have no competing interests.

## Authors' contributions

AJ Wang and MH Chen developed the study hypothesis and designed the study. LS Xiong, SC Liu, PJ Hu and MH Chen revised and reviewed the questionnaire. AJ Wang and XH Liao collected the data. XH Liao, S Peng and YL Xiao performed the statistical analysis. All authors interpreted the data and contributed to drafting of the manuscript. All authors read and approved the final manuscript.

## Pre-publication history

The pre-publication history for this paper can be accessed here:


